# Hydrophobic Drug Carrier from Polycaprolactone-*b*-Poly(Ethylene Glycol) Star-Shaped Polymers Hydrogel Blend as Potential for Wound Healing Application

**DOI:** 10.3390/polym15092072

**Published:** 2023-04-27

**Authors:** Siti Hajar Ahmad Shariff, Rusli Daik, Muhammad Salahuddin Haris, Mohamad Wafiuddin Ismail

**Affiliations:** 1 Department of Chemistry, Kulliyyah of Science, International Islamic University Malaysia, Kuantan 25200, Malaysia; 2 Department of Chemical Sciences, Faculty of Science & Technology, Universiti Kebangsaan Malaysia, Bangi 43600, Malaysia; 3 Department of Pharmaceutical Technology, Kulliyyah of Pharmacy, International Islamic University Malaysia, Kuantan 25200, Malaysia; 4 IKOP Pharma Sdn Bhd, Kuantan 25200, Malaysia

**Keywords:** amphiphilic system, star-shaped polymer, hydrogel, polycaprolactone-*b*-poly(ethylene glycol)

## Abstract

Blending hydrogel with an amphiphilic polymer can increase the hydrophobic drug loading and entrapment efficiency of hydrogel-based formulations. In this study, a hydrogel formulation with star-shaped polycaprolactone-b-poly(ethylene glycol) (PCL-*b*-PEG) as the hydrophobic drug cargo is produced. The 4-arm and 6-arm star-shaped PCL are synthesized with different molecular weights (5000, 10,000, 15,000 g/mol) via ROP and MPEG as the hydrophilic segment is attached via the Steglich esterification. FTIR and ^1^H-NMR analysis showed the presence of all functional groups for homopolymers and copolymers. M_n_ for all synthesized polymers is close to the theoretical value while GPC spectra showed a monomodal peak with narrow molecular weight distribution (PDI:1.01–1.25). The thermal degradation temperature and crystalline melting point of synthesized polymers increase with the increase in molecular weight and number of arms. All formulations possess high drug loading and entrapment efficiency (>99%) and increase with increasing molecular weight, number of arms, and amount of polymer in the formulations. All formulations showed a sustained drug release pattern with no initial burst, which follows the Korsmeyer–Peppas kinetic model. The polymer hydrogel formulations showed antibacterial activity against *E. coli* and *S. aureus*. The hydrogel containing 4-arm PCL_15k_-PEG is chosen as the best formulation due to its high drug release, good antimicrobial activity, and morphology.

## 1. Introduction

Strategic management of chronic wounds is highly needed to reduce casualty numbers. Many researchers have been focusing on dressings that can enhance wound healing throughout the years. The essential characteristics of ideal wound dressings are the provision or maintenance of a moist environment, the improvement of the migration of epidermal cells, the provision of protection against bacterial infection, removability and non-adherence to the wound, non-toxicity, and non-allergenicity [1]. Several wound dressings have been developed based on wound conditions, such as gauze, hydrocolloids, films, hydro fibers, foams, and hydrogels [2].

Hydrogel has been receiving more attention due to its nature, which complies with the ideal wound dressings. Hydrogel has been found to improve the rate of wound healing, as compared with plain gauze, and improve histological outcomes [3]. While various hydrogel-based dressings are available on the market, several limitations have hindered this form of dressing, such as low efficacy towards hydrophobic drug delivery, fast drug release from large porous hydrogels, and low mechanical stability [4]. Therefore, it is crucial to develop a new hydrogel formulation for a wound dressing to overcome these disadvantages.

Hydrogel limits the entrapment of hydrophobic drugs, which is a significant concern since hydrophobic drugs are used extensively in pharmaceutical treatment. Estimation from research shows that approximately 40% of commercial drugs and 60% of compounds in the research and development stage have low water solubility [5]. Another challenge faced by the hydrogel-based dressing is controlling the release rate of drugs according to the patient’s needs. Chronic wounds such as diabetic foot ulcers need a longer time for healing, hence requiring a sustained drug release pattern [6]. In comparison with synthetic hydrophobic polymers, most hydrogel-based systems for wound healing have relatively large pores that cause rapid release of freely encapsulated compounds, limiting their efficacy in drug delivery [7]. Hydrogel has low mechanical stability, which requires adjustment to wound treatment [8]. Hence, hydrogel blending with other polymers can overcome this issue. Nevertheless, this limitation can be overcome by using a polymer-blend system in the hydrogel formulation to optimize drug release based on the drug’s properties, dosage, and pharmacokinetics. Polymer blending is expected to improve a hydrogel’s physical and mechanical properties, release kinetics, biocompatibility, biodegradability, and ability to be processed [9].

The advantages of using polymer-blend systems in controlled-release applications are simple device manufacturing and the ability to control device properties such as hydration and mechanical strength, drug loading, and micro-reservoir size to improve release properties. Hydrophilic polymer attachment to hydrophobic polyester can create an amphiphilic polymeric system with higher hydrophilicity than its homopolymer [10,11,12]. The amphiphilic polymer has a huge advantage since the hydrophobic parts can carry and protect the drugs, while the hydrophilic regions can enhance their stability and solubility in an aqueous medium and water-based hydrogel [13].

Polyester-polyether forms of amphiphilic polymers, particularly polycaprolactone-poly(ethylene glycol), have drawn significant interest due to their usefulness in wound treatment applications and controlled-release systems. The amphiphilic polymer can promote hydrophobic drug entrapment by hydrophobic–hydrophobic interaction with PCL. At the same time, PEG enhances the drug cargo’s solubility for bioapplication [14]. Of particular interest to biomedical applications are biodegradable and biocompatible star-shaped block copolymers with hydrophobic inner parts and hydrophilic outer parts. Drug entrapment can be enhanced by using star-shaped polymers because it gives additional drug-conjugated sites for drug loading [15]. In recent years, the star-shaped drug delivery system has been thoroughly studied for its drug loading capacity in particular and has demonstrated many features in terms of drug entrapment, the pattern of drug release, critical micelle concentrations, and cytotoxicity [16,17].

This research focuses on developing star-shaped PCL-PEG copolymers with different molecular weights and architectures to find the best polymeric system to be incorporated in the hydrogel. Mathematical kinetic analysis of the drug loading and the drug release pattern will provide a novel insight into the production of formulations that offer optimal hydrogels for wound healing applications. This work is expected to solve the limitation of commercialized wound treatment based on hydrogels in terms of the loading and release of drugs. As a result, the efficiency of hydrogels will be increased in terms of better treatment, cost reduction, and time management that will have a significant impact on the human community.

## 2. Materials and Methods

### 2.1. Materials

ε-caprolactone (ε-CL), pentaerythritol, dipentaerythritol, and trifluoroethanol (TFE) was purchased from Merck. Stannous octoate (Sn(Oct)_2_) was purchased from Sigma Aldrich. 4-(dimethyl amino) pyridine (DMAP), 1,3-dicyclohexylcarbodiimide (DCC), Ciproloxacin, and Carbopol 940 used in this study were used without further purification.

Succinilated MPEG (MPEG) with Mn = 5000 Da was synthesized from a previous study by Ismail et al. 2019 [15]. The antimicrobial activity of the hydrogel was tested against Gram-positive Staphylococcus aureus (ATCC 25923) and gram-negative Escherichia coli (ATCC 25922) [18]. All tested strains were purchased from the American Type Culture Collection (ATCC), Manassas, USA.

### 2.2. Synthesis of Star-Shaped Polymers

#### 2.2.1. Synthesis of Star-Shaped PCL

**Figure 1:**
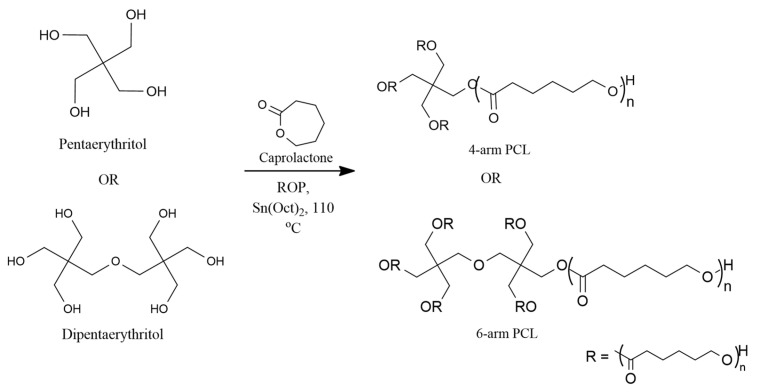
Preparation of 4-arm PCL and 6-arm PCL.

A certain amount of pentaerythritol/dipentaerythritol, Sn(Oct)_2_ (0.1 wt.% of ε-CL) and ε-CL were added into a three-neck round-bottom flask equipped with a reflux condenser under a nitrogen atmosphere according to the molar feed ratio for 4-arm and 6-arm star-shaped PCL. The flask was placed in an oil bath at 110 °C for 24 h with stirring. The reaction was cooled to room temperature, and the product was dissolved in diethyl ether to precipitate the polymer. Figure 1 shows the route of preparation of 4-arm PCL and 6-arm PCL.

#### 2.2.2. Synthesis of Star-Shaped PCL-PEG

**Figure 2:**
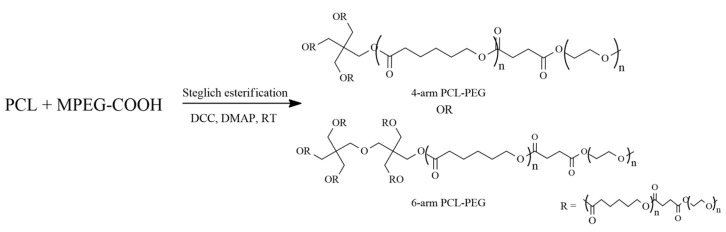
Preparation of 4-arm PCL-PEG and 6-arm PCL-PEG.

The PCL-OH was dissolved in methylene chloride according to the molar feed ratio of 1:4:4:4 and 1:6:6:6 (PCL-OH:MPEG-COOH:DMAP:DCC) for 4-arm and 6-arm copolymers, respectively, and mixed with MPEG-COOH, DMAP, and DCC (Figure 2).

The reaction was carried out at room temperature for 48 h under a nitrogen atmosphere. Dicylcohexylcarbodiurea by-product was removed by filtration. The product was washed and filtered several times to ensure no presence of any unreacted homopolymers.

### 2.3. Hydrogels Formulation

Hydrogel preparation was done according to the method suggested in Jagdale and Pawar [19] with modification. Briefly, PCL-b-PEG polymers were dissolved in TFE for 12 h, followed by ciprofloxacin, and stirred for another 12 h. Carbopol 940 was dispersed in deionized water for 24 h. The drug-loaded solution was added into Carbopol 940 by dropwise addition and was stirred at 2000 rpm for 24 h. The mixture was neutralised by the dropwise addition of TEA. The composition of the hydrogel is listed in Table 1. Formulation A and Formulation B were prepared by varying the composition of the PCL-b-PEG (0.25% and 0.5%).

#### 2.3.1. Synth Drug Loading (DL) and Entrapment Efficiency (EE)

A quantity of 100 mg of the gels was dissolved in a 100 mL phosphate buffer solution (pH 7.4). The solution mixture was shaken for 30 min using a mechanical shaker. A membrane filter was used to filter the mixture into a cuvette. The absorbance of the sample was determined using a UV-Vis spectrophotometer at λ_max_ = 276 nm [20]. The concentration of ciprofloxacin was estimated from the calibration curve using a phosphate buffer as blank [21]. The experiment was performed in triplicate, and the average values were recorded. The drug loading and entrapment efficiency were calculated using Equations (1) and (2) [22].

(1) $$\mathrm{Drug}\;\mathrm{encapsulation}\;\mathrm{efficiency}\;\left(\mathrm{EE}\right)\;\%=\frac{\left(A-B\right)}{A}\times 100$$

where 

A = amount of initial drug in the formulation

B = amount of drug found in solution

(2) $$\mathrm{Drug}\;\mathrm{loading}\;\left(\mathrm{DL}\right)\;\%=\frac{\left(W-w\right)}{W}\times 100$$

where 

W = weight of the hydrogel

w = amount of drug in solution

#### 2.3.2. In Vitro Drug Release

An in vitro drug release study was carried out using the dissolution testing apparatus (basket method). The test was conducted using a 900 mL buffer solution (pH 7.4 ± 0.2) at 100 rpm. A 5 mL sample solution was collected at different time intervals (1, 2, 3, 4, 5, 6, 7 h) and filtered through a membrane. The same volume of fresh dissolution medium at the same temperature was added to substitute for the solution withdrawn after each sampling. The cumulative percentage drug release was calculated [23].

#### 2.3.3. Drug Release Kinetics

The drug release data were applied to zero-order, first-order, Higuchi, Hixson Crowell, and Korsmeyer–Peppas mathematical models. The parameters of the models were obtained by linear regression. The model with the highest regression coefficient (R2) was chosen as the best fit to determine the release kinetics of the drug from the formulations [24].

#### 2.3.4. Statistical Analysis

The study results were demonstrated as a mean of triplicate data (±SD). The results were analyzed using GraphPad Prism 9.0. One-way analysis of variance (ANOVA) was used with a pairwise comparison based on the *t*-test procedure to compare Formulation A and Formulation B. The differences in the cumulative percentage of drug release were considered significant at a *p* value < 0.05.

#### 2.3.5. Antimicrobial Assay

The in vitro antimicrobial assay of the formulations with the highest drug release was evaluated using agar well diffusion methods. The microbial suspension was spread onto the surface of agar plates using sterile cotton swabs. Then, a sterile pipette tip was used to make wells (5 mm) on the agar plates. The formulations were weighted to approximately 5 ± 1 mg and were placed into each well. The inhibition zone around the drug-incorporated hydrogel was compared after 12 and 24 h of incubation at 37 °C. The average diameter was measured in millimetres, with hydrogel without drug as a control. The experiment was done in triplicate for each formulation [25].

### 2.4. Instrumentations

FTIR spectra were recorded with the FTIR-ATR Perkin–Elmer spectrometer in the region 4000–400 cm^−1^. Proton NMR (^1^H-NMR) spectra were recorded on a Bruker Ultra Shield Plus at 500 MHz with tetramethyl silane (TMS) as the internal reference. GPC analysis was performed using Waters 2414 Gel Permeation Chromatography. Thermogravimetric TGA/DTG analysis was carried out using a Mettler Toledo Thermogravimetric Analyzer. DSC analysis was carried out using a Mettler Toledo Differential Scanning Calorimeter under nitrogen with a flow rate of 10 mL/min and a heating rate of 10 °C/min. The pH of the gel formulations was determined using a Mettler Toledo digital pH meter. The viscosity of the gel formulations was determined using a Brookfield viscometer with spindle no. 64 at 50 rpm [26]. The hydrogel was lyophilized using a freeze dryer at a temperature of −55 °C and a pressure of 30 Pa for 48 h to remove any remaining solvents. The freeze-dried hydrogel was then subjected to SEM analysis.

## 3. Results and Discussion

### 3.1. Synthesis of Star-Shaped PCL

Three 4-arm and 6-arm PCL with different molecular weights were obtained using different monomer:initiator ratios. The temperature of 110 °C was selected as it was the optimised temperature for ROP of ε-CL with Sn(Oct)_2_ as the catalyst [27,28]. A higher temperature promotes intermolecular and intramolecular esterification that broadens the molecular weight distributions of the synthesized polymers [29].

The FTIR spectra show the presence of a sharp and intense band around 1720 cm^−1^ for both 4-arm and 6-arm PCL, indicating the appearance of carboxylic ester (C=O) groups for the repeating units of the PCL chain (Figures S1 and S2). The C-H band of PCL methylene group backbone appeared around 2980 cm^−1^ to 2850 cm^−1^ for both 4-arm and 6-arm PCL. The presence of these bands indicates the successful formation of PCL via ROP of cyclic ε-CL. The C-O stretch band for both 4-arm and 6-arm polymers appeared around 1190 cm^−1^. The hydroxyl functional group band appeared around 3500 cm^−1^ and 3400 cm^−1^ with low intensity due to the low -OH group per PCL chain [30].

In the ^1^H-NMR spectra of the synthesized 4-arm and 6-arm PCL with different molecular weights (Figures S3 and S4), the signal for the methylene proton (a) of the pentaerythritol appeared around 4.10 ppm. The chemical shifts (b–e) appeared around 1.30 ppm to 4.10 ppm (δ 1.3 and δ 1.6, δ 2.2, δ 4.0, (*m*, -CH_2_-), (*t*, -CH_2_-), (*t*, -CH_2_-O-)) corresponded to the PCL backbone, indicating the successful occurrence via ROP of ε-CL. The methylene proton (f) peak was observed around 3.60 ppm (*t*, -CH_2_-OH), indicating the terminal group of the PCL. The core arms had a similar -OH terminal group as the active site that can initiate the ROP of the cyclic ε-caprolactone [31].

### 3.2. Synthesis of Star-Shaped PCL-PEG

The coupling reaction between the star-shaped PCL and succinylated MPEG was done via the Steglich esterification reaction. The FTIR spectra (Figures S5 and S6) show that the sharp C=O stretching band around 1725 cm^−1^ was the carbonyl group of the ester linkage. The appearance of this band indicates the successful coupling reaction between PCL and PEG [32]. Meanwhile, the sharp and intense bands that appeared around 1108 cm^−1^ to 1106 cm^−1^ correspond to the C-O functional group of the PEG ether polymer backbone. The C-H stretching bands for the antisymmetric and symmetric vibrations in the PEG and PCL repeating chain segments appeared around 2940 cm^−1^ to 2880 cm^−1^. The absence of the -OH band confirmed the coupling reaction of PCL and MPEG-COOH.

As for proton NMR for 4-arm and 6-arm PCL-PEG copolymers (Figures S7 and S8), the chemical shifts for repeating units of the PCL backbone appeared around 1.4 ppm (*m*, 2H, -CH_2_-), 1.7 ppm (*m*, 2H, -CH_2_-), and 2.3 ppm (*t*, 2H, -CH_2_-). A triplet around 4.1 ppm (*t*, 2H, -CH_2_O-) denotes the appearance of methylene ester for the PCL polymer. Meanwhile, the chemical shifts between 3.6–3.8 ppm (*m*, -OCH_2_CH_2_OCH_3_-) and 3.4 ppm (*s*, -OCH_3_-) denote the repeating unit of PEG and methylene end group of MPEG, respectively. The successful conjugation of the PCL and PEG blocks was signified by the presence of a triplet around 2.8 ppm (*t*, -COCH_2_CH_2_CO-) for the methylene group ester linkage between PCL and PEG. Apart from that, the absence of the hydroxyl end group for the homopolymer PCL peak confirmed the formation of PCL-PEG copolymer [33].

### 3.3. Molecular Weight Analysis

The molecular weights of the synthesized star-shaped polymers were determined using ^1^H NMR and GPC analysis. The degree of polymerization for the PCL repeating chains was calculated from the integration ratio of the methylene protons for the repeating units at around 2.2 ppm and protons for the terminal unit at around 3.6 ppm based on the ^1^H-NMR spectrum [34]. The M_n_ obtained from the synthesized polymers was close to the theoretical value (Table 2).

The star-shaped copolymers have a well-defined 4-arm and 6-arm structure due to the same hydroxyl active site in the homopolymer PCL [35]. Theoretically, an MPEG segment will attach to each PCL arm; a ratio of the MPEG signal to PCL signal was used to calculate the average molecular weight (M_n_) of the block copolymers. DP_arm_ was obtained by comparing the ratio of integration area for the peak f at around 2.3 ppm of the PCL backbone with the peak h of the PEG end group at around 3.4 ppm [36]. The M_n_ of the copolymers obtained was comparable to the theoretical M_n_ value (Table 3).

The M_n_ value obtained from the GPC analysis was comparably different from the theoretical value. These results corresponded with the study conducted by Mortazavian et al. [37] and Yan et al. [38], where they reported a significantly different M_n_ value obtained from GPC analysis compared with the the theoretical and ^1^H-NMR values for the synthesized star-shaped polymers. The GPC column separates samples based on the hydrodynamics size and not molecular weight. The analysis was based on the theory that a specific size in the solution correlates to a specific molecular weight given in the calibration. The retention time depends on that molecular size [39]. In a solution, polymers with identical molecular weights but different architecture can coil up to form a sphere with the same hydrodynamic volume. However, their molecular weight can vary from one to another, although having the same volume [40]. Moreover, the molecular weight of the star-shaped polymer in GPC analysis is expected to have a smaller hydrodynamic volume than its linear counterpart [38,41].

**Figure 3:**
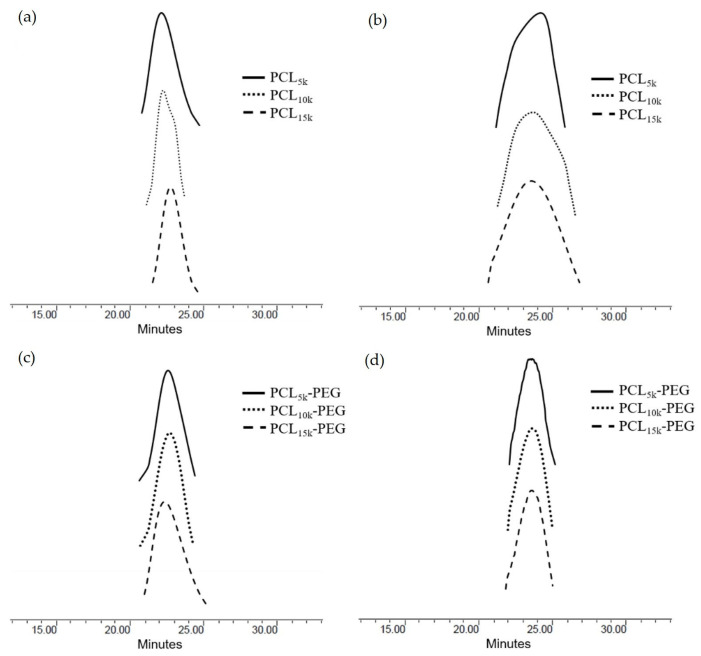
GPC chromatogram of (**a**) 4-arm PCL, (**b**) 6-arm PCL, (**c**) 4-arm PCL-PEG, and (**d**) 6-arm PCL-PEG.

Although the molecular weight analysis using GPC is different from ^1^H-NMR, GPC analysis is necessary to define the molecular weight distribution of polymers. Molecular weight distribution can influence polymeric properties such as viscosity, processability, and crystallization. Minor variations in the distribution might considerably impact the polymer’s content, resulting in significant differences in the properties [42]. A narrow distribution with a polydispersity index (PDI) less than 1.3 in star-shaped polymers is desirable since it signifies a controlled polymerization reaction on each polymeric arm and symmetrical peaks [43]. The PDI obtained from the GPC analysis (Table 3) was narrow (PDI ≈ 1.01–1.20), indicating monodispersity. Therefore, it can be said that the polymerization process of ε-CL was conducted under a controlled condition. This result is further proven by observing the GPC chromatogram (Figure 3) for the star-shaped PCL and PCL-b-PEG, whereby the GPC curves show a unimodal peak for all polymers. The unimodal peak indicates monodispersity of the molecular weight and the absence of homopolymer PCL and MPEG monomers [44]. This is due to the minimal transesterification or backbiting during the copolymerization reaction, which resulted in the symmetrical polymerization in each arm [45].

### 3.4. Thermal Analysis

All polymers had high thermal stability based on the initial thermal degradation temperature (T_d_, onset), around 343.6–374.3 °C (Table 4 and Table 5). The main thermal degradation temperature (T_d_, max) increased with the increasing molecular weight of the homopolymer PCL. Therefore, more energy was needed to break the polymer chain [46]. However, the overall T_d_, the max temperature of 6-arm PCL, was slightly lower than its 4-arm PCL analogue. This is due to the higher branching of the 6-arm PCL. Since the thermal stability of polymers is heavily reliant on their chemical structure and molecular weight, an increase in branching decreases the polymer chain. Consequently, there is reduced cross-linking and degree of entanglement, hence less energy is required to break the ester linkage of the PCL backbone.

Meanwhile, the addition of MPEG increased the overall thermal stability of the block copolymers compared with the homopolymer PCL. The MPEG modified the -OH end group susceptible to faster thermal degradation to a more stable -OCH_3_ group [47]. Homopolymer MPEG has the highest thermal degradation temperature compared with homopolymer PCL and block copolymers PCL-PEG. The increase in the thermal stability demonstrated synergistic thermal stability and the compatibility of the block copolymers [48,49]. Compared with 4-arm PCL-PEG, 6-arm PCL-PEG has a higher T_d_ and onset temperature, and contains a higher PEG segment; therefore, more energy is required to initiate the degradation reaction. However, 6-arm PCL-PEG has a lower T_d_ and max temperature than 4-arm PCL-PEG, also due to the higher PEG segments. The 6-arm PCL-PEG have more hydrophilic regions that lead to a rapid hydrolytic degradation to produce free OH^−^, which later become a catalyst for the breakage of the ester linkage and accelerate the degradation of the copolymers [50].

**Figure 4:**
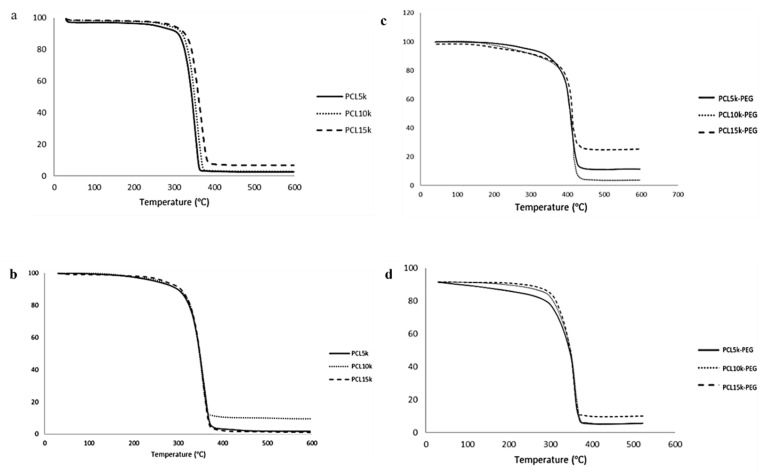
(**a**) TGA of 4-arm PCL, (**b**) TGA of 6-arm PCL, (**c**) TGA of 4-arm PCL-PEG, and (**d**) TGA of 6-arm PCL-PEG.

All synthesized polymers showed a single step-degradation (Figure 4). Single-step degradation indicates that the copolymers are compatible with each other as block copolymers [51] and only one decomposition process due to the homogeneous materials [52].

**Figure 5:**
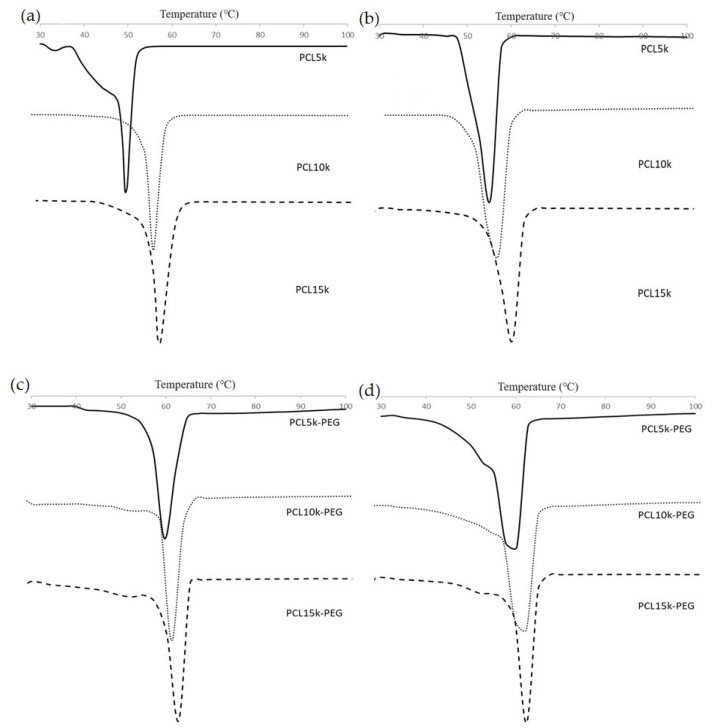
DSC thermogram of (**a**) 4-arm PCL, (**b**) 6-arm PCL, (**c**) 4-arm PCL-PEG, and (**d**) 6-arm PCL-PEG.

Homopolymer PCL has a T_m_ within the melting range of PCL, which is around 60 °C [53] (Figure 5). The melting point of the synthesized 4-arm and 6-arm PCL increased with M_n_ due to the arm’s length [54]. The melting point of a crystal is determined by the strength of the molecule’s hydrogen bonds, the effects of molecular shape, size, and symmetry on crystal packing, and other factors such as charge transfer and dipole–dipole interactions [55]. The increasing molecular weight of PCL (arm lengths) limits free molecular motion through crystallization and improves molecule packing in crystals, making them harder to rearrange to form lamellae regions [56]. As a result, the higher molecular weight requires more energy absorption to melt the crystals [57]. Apart from that, it can also be observed that the T_m_ of the homopolymer PCL increases with the increasing number of arms. This might be due to the increase in the compactness of the structure as polymerization of PCL occurred, which reduced the molecular surface area per atom. Consequently, it increases the stability of the polymer, making it hard to break the compact structure [58,59].

The same trend is observed on the block copolymers PCL-PEG. The addition of PEG increases the crystalline melting temperature of the copolymers due to the interaction between the melting of PCL and PEG blocks [36]. The temperature is close to the melting point of PEG because PEG has a higher T_m_ than PCL. Apart from that, 6-arm PCL-PEG has a higher crystalline melting point compared with 4-arm PCL-PEG. The different number of arms results in different PEG added to each PCL arm. The addition of PEG increases the polymer chain mobility that allows chain rearrangement [60,61]. PEG promotes easier rearrangement to form a more stable crystalline structure. Hence, more energy is needed to melt the crystalline structure and free the polymer chains of the 6-arm PCL-PEG [60,62].

The monomodal exotherm peak indicates that the PCL homopolymers can crystallize completely and give crystals when quenched from the melt. Meanwhile, the monomodal peaks of the PCL-PEG copolymers might come from the crystalline domain of the dominant PEG segment, making the melting temperature of PCL blocks impossible to measure individually because of inadequate phase separation and improved phase compatibility between PCL and PEG blocks. A certain amount of PEG can stimulate the crystallization of block copolymers as it can move freely compared with the rigid PCL core [63]. This shows that the block copolymers are comprised of semicrystalline PEG segments and PCL blocks scattered within the crystalline PEG phase [64].

### 3.5. Hydrogel Formulations

Twelve hydrogel formulations were prepared by varying the composition of the 4-arm and 6-arm PCL-PEG to observe the effect of different polymer compositions on the properties of the formulations. Amphiphilic PCL-PEG was chosen to ensure homogenous hydrogel formation [65]. The PCL as the inner segment encapsulates hydrophobic ciprofloxacin model drugs. Meanwhile, the PEG as the outer segment increased the polymer’s solubility in hydrogel formulation to form a homogeneous formulation. Carbopol 940 was selected as the gelling agent due to its simplicity to be developed at room temperature and its large viscosity range, which made it easier to modify the physical properties of the gel [66]. Carbopol also possesses high thixotropy, a rheological characteristic referring to changes in viscosity with the time of application, which was desirable for topical gel [67]. Compared with other gelling agents such as Carbopol 934 and Ultrez 10, Carbopol 940 was found to have superior flow-modifying properties for topical delivery and a greater effect in extending drug release from hydrogel formulations compared with Carbopol 934 [68,69]. Additionally, Carbopol 940 was reported to show no obvious toxicity to the target organs and tissues during long-term repeated treatment for skin wound healing [70]. The amount of Carbopol 940 used was kept to 0.5% *w*/*w* to get excellent skin feel for topical application [71]. Ciprofloxacin was selected as a hydrophobic model drug with antimicrobial properties. The ciprofloxacin model drug used was limited to a minimum amount that demonstrated its efficacy [72,73].

After complete dispersion of the hydrogel components, TEA was added to the homogenous formulation to alter the pH value and thicken the hydrogel. Carbopol was then able to absorb and retain water, making the crosslinking chains to hydrate. As a result, the chains partially uncoiled due to the electrostatic repulsion to form a gel network with stronger bonds [74,75]. The formulations obtained had an opaque white appearance with good homogeneity, indicating good hydrogel characteristics (Table 6) [76]. After three weeks of studies, observation on the hydrogel formulations showed no physical changes in the colour and homogeneity, indicating the stability of the formulations.

The viscosity of the hydrogel formulations was high, with a value above 10,000 cP. Previous studies showed that gels with high viscosity have high consistency and good adherence to the skin [67]. One of the essential physical characterizations of the hydrogel formulations is viscosity because it contributes to the properties of the formulations, such as spreadability, pourability, and consistency [77]. Higher gel viscosity also prolonged the release of the drug, improving patient compliance with treatment due to a reduced number of applications [78].

The overall trend for the viscosity shows that Formulation B has a higher viscosity than A for all formulations. The viscosity depends on the concentration (%) of the polymer in the solution and the molecular weight. Hence, a greater polymer concentration resulted in a higher viscosity of the solution [79]. Meanwhile, the same increasing trend was found when comparing the viscosity between formulations with 4-arm PCL-PEG (F1–F3) and formulations with 6-arm PCL-PEG (F4–F6). The increase in the viscosity was associated with the increase in the overall molecular weight of the star-shaped polymers [65]. First, the higher viscosity of the formulations was due to the higher molecular weight of PCL. Second, as the number of arms increased, the amount of PEG segments attached to each arm also increased. Therefore, even though 4-arm and 6-arm homopolymer PCL have the same molecular weight, the addition of the PEG segment to each arm makes the overall molecular weight of 6-arm PCL-PEG higher than that of 4-arm PCL-PEG. Bigger polymer molecules possess more intermolecular interaction, and hence increase the viscosity [80].

Apart from viscosity, pH value also plays a crucial role in the properties of the gel. Alkaline formulations with a pH >8 will agglomerate and become too concentrated, affecting the spreadability on human skin, while acidic formulations with pH <5 dilutes the formulation and irritates the skin [81]. The optimum viscosity of Carbopol for good spreadability is typically achieved at a pH range of 6.5–7.5 [82]. Therefore, the pH value of the formulation was adjusted to the range between 7.0–7.4, which was in the optimum range and suitable to be used on human skin [26,83]. There were no significant changes in the pH of the formulation after several weeks of studies, indicating good stability and long lifespan of the formulations [84].

### 3.6. Drug Loading (DL) and Entrapment Efficiency (EE)

The drug was incorporated into the hydrogels via in situ loading. The polymer precursor solution was mixed with a drug-loaded polymer, and the hydrogel network formulation and drug entrapment were achieved simultaneously [85]. Based on calculated values, both formulations showed high drug entrapment efficiency (EE) and drug loading (DL) with a value >99%. This proves the role of the PCL core as a drug cargo to enhance the capability to load hydrophobic drugs, which was due to the hydrophobic–hydrophobic interaction between ciprofloxacin and the PCL block [86]. This result was consistent with the literature, where PCL-PEG micelles favored the hydrophobic atorvastatin drug than the hydrophilic rosuvastatin drugs [87].

There are no significant differences in the EE and DL of all the formulations, signifying the amount of the copolymers used, and the number of polymeric arms did not significantly enhance the loading capacity. However, the overall trend for the drug loading and entrapment efficiency is that Formulation B has a slightly higher DL and EE than Formulation A. A higher concentration of star-shaped PCL-PEG increased the opportunity for a higher amount of polymer for drug entrapment [88]. Apart from that, the polymer concentration enhances the viscosity of the gel, increasing the diffusional resistance of the drug molecules. Consequently, more drug is entrapped in the cargo as the drug diffusion out of the polymeric cargo is reduced due to a longer diffusional path [89]. The same observation has been reported in the study conducted by Huang et al. [90].

The DL and EE of the formulations are also affected by the molecular weight of the polymers, where higher molecular weight polymers have a slightly higher DL and EE. As the molecular weight of the polymer increased due to the longer PCL segment, the hydrophobic-hydrophobic interaction between ciprofloxacin and the PCL also improved, thus allowing higher drug loading in the formulation. Meanwhile, the attachment of the PEG segment increases the overall molecular weight of the star-shaped PCL-PEG, giving the polymer a higher degree of entanglement. This reduces the drug’s molecular diffusion area and permeation across the matrix gel [91].

Star-shaped PCL-PEG copolymers offer additional drug-conjugated sites and looser space for drug loading. The high number of arms affects the drug loading and entrapment capability [92], as it enhances the DL and EE due to the larger sites that can act as a drug reservoir. Therefore, compared to 4-arm PCL-PEG, 6-arm PCL-PEG with more hydrophobic arms in the polymers, led to bigger cores that easily encapsulated the hydrophobic drugs.

### 3.7. Drug Release Analysis

The release of the drug loaded via in situ loading is determined by diffusion, hydrogel swelling, reversible-polymer interaction, or the degradation of labile covalent bonds [93]. Due to the prolonged degradation rate of PCL, the formulations containing 4-arm and 6-arm PCL-PEG in this study were expected to exhibit a controlled release behavior via diffusion-controlled release [94]. The drug began to release from the surface of the sphere and then continued to release from the inner layers of the sphere [95]. Since the ciprofloxacin was encapsulated in the PCL inner segment of the star-shaped PCL-PEG instead on the surface of the system, this resulted in slower drug release with no initial burst rate.

The drug release of Formulation B is higher compared with that of Formulation A (Table S1) for the hydrogel containing 4-arm PCL-PEG (F1–F3). Higher DL and EE in Formulation B than A leads to a higher amount of drug released per unit area exposed to the surface of the polymer matrix [96]. Additionally, increasing the molecular weight of the polymers also enhanced the DL and EE of the formulations, hence the higher the drug release. This result corresponded with the study done by Younis et al. [97].

However, as the polymer concentration in the formulations increases, the viscosity of the polymer gel is enhanced. Subsequently, the density of the polymer matrix and diffusion path length that the drug molecules have to cross becomes greater. Therefore, the drug diffusion coefficient across the hydrogel matrix is decreased [89,96]. This can be seen in the drug release pattern of formulations containing 6-arm PCL-PEG (F4–F6), where Formulation A has higher drug release than Formulation B due to its lower viscosity. Apart from that, formulations containing 4-arm PCL-PEG have a higher release rate than those containing 6-arm PCL-PEG. This might be due to its lower molecular weight, which makes them possess a high elastic modulus. Thus, the matrix becomes more deformable, causing the pores to expand due to osmotic pressure [98]. In addition, the blending of hydrophilic PEG into the hydrophobic PCL can increase pore formation and increase the rate of polymer degradation, leading to faster drug release [99].

**Figure 6:**
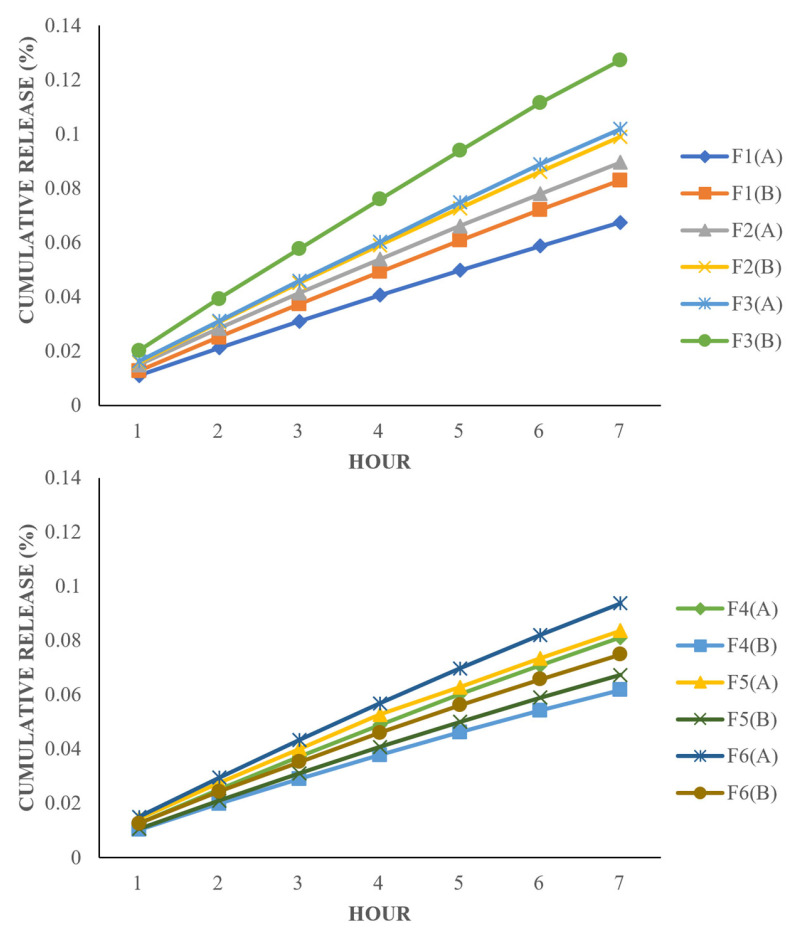
Graph of cumulative release (%) of the polymeric hydrogel formulations.

The percentage of drug release from the formulations was low across the 7 h of the study (Figure 6). At 7 h, the highest drug release was only about 25% of the total encapsulated drug. This is related to the presence of PCL, which influenced the hydrophobic–hydrophobic interaction between the model drug and PCL. The low rate occurred because the release behavior is not severely affected by the degradation of PCL blocks, as the PCL degradation rate is very slow in an aqueous medium due to crystalline and hydrophobic properties. Hence, the release of ciprofloxacin was facilitated by the penetration of water into the amorphous region of the polymer matrix that diffuses out of the matrix together with the drug [100]. The same trend was reported in the study conducted by Kheiri Manjili et al. [101] and Zamani et al. [102].

### 3.8. Drug Release Kinetic Study

The development of mathematical modelling requires knowledge of all phenomena influencing drug release kinetics which has a significant value in the formulation optimization [103]. Various mathematical kinetic models (Zero-order, First-order, Higuchi, Hixson–Crowell, and Korsmeyer–Peppas) were used to examine the most suitable dissolution profile for the polymeric hydrogel formulations, which is the main fitting model for hydrogel-based drug delivery systems [104].

The zero-order model describes drug release that is independent of the drug concentration. Conversely, the first-order model corresponds to a drug release that is dependent on the drug concentration. The Higuchi model explains the diffusion process for the release of low-solubility drugs distributed within an insoluble and swellable porous matrix based on Fick’s diffusion law. The Hixson–Crowell equation defines drug release from dosage forms with variable diameter and surface area. Meanwhile, Korsmeyer–Peppas explains a mixed release mechanism that is linked to a number of factors such as polymer swelling, polymer erosion, matrix porosity, and drug diffusion rates for drugs in swelling systems [104,105]. The best linearity for all formulations was obtained from the values in the Korsmeyer–Peppas model (Table 7). Hence, Korsmeyer–Peppas is the best-fitted mathematical modell for polymeric hydrogel formulations. This result is similar to those of previous studies that also reported the same drug release kinetics for the polymeric hydrogel drug release systems [105,106].

The data from the in vitro drug release studies were plotted as cumulative drug release (%) versus the log of time. Then, the *n* value was obtained from the slope of the graph to characterize different release mechanisms for the matrices [107]. Based on the results, the *n* value of the formulations was higher than 0.89 (*n* > 0.89), indicating a Case II transport mechanism [108]. This mechanism showed that the drug release is by both diffusion and relaxation of the polymer chain, which might be due to the chain disentanglement and swelling of the hydrophilic polymer, such as Carbopol, that can initiate water diffusion. Apart from that, the higher number of hydrophilic PEG segments in each arm of the star-shaped PCL-PEG can also facilitate water diffusion into the formulation compared with the PCL segment [99]. This proved that the drug release of ciprofloxacin from the PCL-PEG hydrogel was indeed facilitated by the penetration of water into the polymer matrix. The mechanism also describes the effect of polymer hydration and swelling behavior on drug release for polymeric and swellable systems. The drugs probably diffuse out of an outer gel layer that erodes, releasing the polymers containing the drugs into the aqueous medium [104].

### 3.9. Statistical Analysis

Statistical analysis was carried out to analyze whether significant differences can be reported in the drug release of all the formulations. The treatment (between columns) effect measures the average difference between subjects given a particular treatment and the overall mean [109]. The formulations were subjected to 7 h of dissolution study. The results showed that the percentage of drug release at each time point using ANOVA was statistically significant with a *p* value < 0.05. This indicates that the drug release profile depends on the drugs’ dissolution behavior and release pattern from the hydrogel. In this study, drug release is affected by the diffusion and relaxation of the hydrophilic polymer chain. Therefore, drug release may be enhanced in a time-dependent way by tailoring the composition of the hydrophilic polymers in the hydrogel formulations [110].

Then, *t*-test analysis was used to compare the significant difference between Formulation A and Formulation B for F1–F6. Based on the result, Formulation A was significantly different from Formulation B for F1–F6 (*p* value < 0.05), indicating the effect of different polymers’ concentrations (%) on drug release. Apart from that, F3 and F6, which possessed the highest drug release percentages in the formulations containing 4-arm and 6-arm PCL-PEG, respectively, were also compared to observe whether they were statistically different from each other. Based on the result, F3 and F6 were significantly different in the effect of the formulations’ compositions towards drug release with a *p* value < 0.05. As such, F3 and F6 were chosen for further analysis in this study.

### 3.10. Antimicrobial Activity

The antimicrobial activity was performed on four formulations with the highest drug release rate. Since the release rate is significantly affected by the polymers’ composition, the formulations with the highest polymer composition (F3 and F6) were selected to represent both 4-arm and 6-arm PCL-PEG, respectively. The in vitro study measured the diameter of zones of inhibition for each formulation and compared them to the control. Staphylococcus aureus was chosen as the Gram-positive bacteria (Figure S9), and Escherichia coli was selected as the Gram-negative bacteria (Figure S10). These bacteria were selected as they were among the most frequently isolated pathogens from wound infections [111,112].

Based on the antimicrobial study (Table S2), the formulations containing ciprofloxacin model drugs (F3 and F6) showed activity against both Gram-positive and Gram-negative bacteria. Meanwhile, no inhibition zones were observed for STDF3 and STDF6 since both formulations did not contain ciprofloxacin to inhibit the bacteria. As such, no antibacterial activity was observed, indicating that the inhibition zone occurred due to the release of the ciprofloxacin model drug from the PCL-PEG hydrogel formulations.

There were no inhibition zones observed during the first hour for both F3 and F6, showing no initial burst of the drug released from the drug cargo. After 12 h of observation, F3 had higher antibacterial activity than F6. Meanwhile, after 24 h, it could be seen that the inhibition zone for both formulations doubled compared to the 12 h incubation zone, indicating a controlled ciprofloxacin drug release from the polymeric hydrogel formulations. Apart from that, the inhibition zone for F3 was higher compared with that of F6 for both bacteria, due to its higher drug release rate. The highest inhibition zone for both formulations after 24 h of incubation was *E. coli*. This result was expected since ciprofloxacin was highly effective against Gram-negative bacteria, especially Enterobacteriaceae such as *E. coli*, compared to Gram-positive bacteria [113]. Additionally, *E. coli* possessed an abundance of outer membrane proteins called porins that increased the sensitivity of *E. coli* towards ciprofloxacin [114]. These results correspond with past studies that reported the sensitivity of these bacterial strains against ciprofloxacin [114,115]. These results indicate the capability of the PCL-PEG polymeric hydrogel blends to transport hydrophobic drugs. Modifications to the water-based hydrogel enabled the hydrophobic–hydrophobic interaction within the system for effective delivery towards wound healing applications.

### 3.11. Morphological Analysis

**Figure 7:**
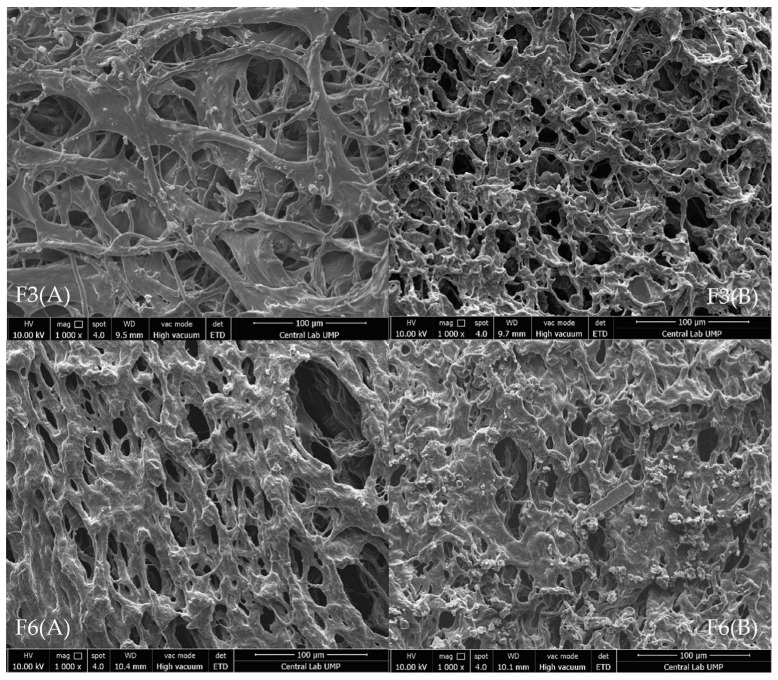
SEM images of the polymeric hydrogels.

The F3 and F6 formulations were subjected to SEM analysis to study morphology and its possible effect on the release rate of the polymeric hydrogels. The analysis shows that the formulations possessed an entangled irregular crosslinking network (Figure 7). A porous structure was observed on all formulations, indicating a high degree of swelling of the developed gels. This is a desirable structure as it affects the amount of water penetration into the hydrogel network [116]. In addition, pore structures affect the properties of hydrogels, such as swelling rate, mechanical strength, and degree of sensitivity [117].

Based on SEM analysis, F6 has smaller pores compared with F3 due to the higher PEG composition and viscosity, making the gel more compact. The hydrogels with denser and tighter networks hinder the polymer chain’s mobility and minimize the water uptake capacity, lowering the swelling of the hydrogels [118]. Consequently, it reduces the drug release rate of the formulations as the release mechanism depends on the water diffusion and swelling behavior.

Additionally, Formulation A has larger pores compared with Formulation B, which increases the release rate of the formulations. Therefore, F6 (A) has a higher release rate than F6 (B). However, the data show that F3 (B) has a higher release rate than F3 (A). Since ciprofloxacin is already loaded into the PCL-PEG during preparation, this causes in the underutilization of the total pore volume. As a result, the release rate depends on the drug loading capacity [119]. These results corresponded with the drug release and antimicrobial study of the hydrogels.

## 4. Conclusions

Star-shaped polymers with different numbers of arms and average molecular weight for polymeric hydrogel drug delivery systems were successfully synthesized and characterized. The polymeric hydrogel formulations F1–F6 (A and B) were prepared using Carbopol as the gelling agent. All formulations showed a high DL and EE with a value >99%. The DL and EE of ciprofloxacin increased with the increasing number of arms, molecular weight, and concentration of the polymers.

The drug release analysis showed different release patterns between the formulations containing 4-arm and 6-arm PCL-PEG. For formulations that contain 4-arm PCL-PEG, Formulation B had a higher drug release than A. Meanwhile, the opposite trend was observed for the formulations containing 6-arm PCL-PEG. The highest drug release was exhibited by F3 (B) with 0.50% (*w*/*w*) of 4-arm PCL15k-PEG. This result proved that the hydrophobic drug release was affected by the concentration of polymer used in the formulations.

The synthesized 4-arm and 6-arm PCL-PEG can be incorporated into hydrogel formulations, and the drug loading and release of the formulations are affected by the presence of the star-shaped PCL-PEG with different architectures and molecular weights.

## Figures and Tables

**Table 1:** Hydrogel composition (*w*/*w* %).

Ingredients	Formulation A (% *w*/*w*)	Formulation B (% *w*/*w*)
PCL-*b*-PEG	0.25	0.5
Ciprofloxacin	0.3	0.3
Carbopol 940	0.5	0.5
Methyl paraben	0.08	0.08
Propyl paraben	0.02	0.02
Trifluoroethanol	33.0	33.0
Deionized water	Up to 100	Up to 100
Triethylamine	q.s	q.s

q.s—quantum satis.

**Table 2:** Molecular weight characteristics of 4-arm and 6-arm PCL.

Sample	^a^ M_n_, (g/mol)	^b^ DP_PCL_	^b^ M_n_, (g/mol)	^c^ M_n_, (g/mol)	^c^ PDI
4-arm PCL_5k_	5100	44	5300	6500	1.01
4-arm PCL_10k_	10,100	84	9800	13,200	1.11
4-arm PCL_15k_	15,100	128	14,800	18,400	1.09
6-arm PCL_5k_	5300	42	5200	9600	1.25
6-arm PCL_10k_	10,200	84	10,100	13,400	1.21
6-arm PCL_15k_	15,300	126	14,800	20,200	1.07

^a^ Determined from monomer feed ratio, ^b^ determined from ^1^H-NMR, ^c^ determined from GPC.

**Table 3:** Molecular weight characteristics of 4-arm and 6-arm PCL-PEG.

Sample	^a^ M_n,_ (g/mol)	^b^ DP_PCL_	^b^ M_n,_ (g/mol)	^c^ M_n_, (g/mol)	^c^ PDI
4-arm PCL_5k_-PEG	25,900	44	27,600	18,600	1.05
4-arm PCL_10k_-PEG	30,900	84	30,400	25,200	1.04
4-arm PCL_15k_-PEG	35,900	128	36,300	33,300	1.06
6-arm PCL_5k_-PEG	36,500	42	38,700	28,600	1.03
6-arm PCL_10k_-PEG	41,400	84	41,700	32,700	1.05
6-arm PCL_15k_-PEG	46,500	126	48,000	38,500	1.05

^a^ Determined from monomer feed ratio, ^b^ determined from ^1^H-NMR, ^c^ determined from GPC.

**Table 4:** TGA results for succinylated MPEG and homopolymer PCL.

Sample	T_d, onset_ (°C)	T_d, max_ (°C)
MPEG-COOH	387.8	422.1
4-arm PCL_5k_	326.3	361.9
4-arm PCL_10k_	331.2	369.4
4-arm PCL_15k_	339.3	380.6
6-arm PCL_5k_	327.6	367.2
6-arm PCL_10k_	329.7	368.3
6-arm PCL_15k_	331.5	372.2

**Table 5:** TGA results of the synthesized PCL-PEG.

Sample	T_d, onset_ (°C)	T_d, max_ (°C)
4-arm PCL_5k_-PEG	365.7	406.1
4-arm PCL_10k_-PEG	366.9	407.8
4-arm PCL_15k_-PEG	369.2	408.3
6-arm PCL_5k_-PEG	367.3	405.0
6-arm PCL_10k_-PEG	374.3	405.5
6-arm PCL_15k_-PEG	375.5	407.3

**Table 6:** Data on the physical characterization of the polymeric hydrogel formulations.

Formulations		Opacity	pH	Viscosity (cP)
F1 (4-arm PCL_5k_-PEG)	A	Opaque	7.36 ± 0.01	10,600 ± 0.02
	B		7.39 ± 0.02	10,800 ± 0.02
F2 (4-arm PCL_10k_-PEG)	A	Opaque	7.32 ± 0.02	10,700 ± 0.03
	B		7.28 ± 0.02	10,800 ± 0.02
F3 (4-arm PCL_15k_-PEG)	A	Opaque	7.41 ± 0.04	10,800 ± 0.04
	B		7.32 ± 0.01	10,900 ± 0.03
F4 (6-arm PCL_5k_-PEG)	A	Opaque	7.26 ± 0.02	10,500 ± 0.02
	B		7.44 ± 0.02	10,900 ± 0.01
F5 (6-arm PCL_10k_-PEG)	A	Opaque	7.35 ± 0.02	11,200 ± 0.02
	B		7.34 ± 0.02	11,200 ± 0.01
F6 (6-arm PCL_15k_-PEG)	A	Opaque	7.43 ± 0.02	11,300 ± 0.01
	B		7.43 ± 0.02	11,500 ± 0.02

**Table 7:** Diffusion exponent value (*n*) based on the Korsmeyer–Peppas Kinetic model.

Formulations		Coefficient, R^2^	*n*
F1	A	0.9999	0.9340
	B	0.9999	0.9641
F2	A	1.0000	0.9234
	B	0.9999	0.9442
F3	A	1.0000	0.9494
	B	0.9999	0.9492
F4	A	0.9997	0.9536
	B	0.9996	0.9293
F5	A	0.9992	0.9106
	B	0.9996	0.9506
F6	A	0.9997	0.9428
	B	0.9997	0.9172

## Data Availability

The data presented in this study are available in article and supplementary material.

## Supplementary Materials

The following supporting information can be downloaded at: https://www.mdpi.com/article/10.3390/polym15092072/s1, Figure S1: FTIR spectra of 4-arm (a) PCL5k (b) PCL10k and (c) PCL15k; Figure S2: FTIR spectra of 6-arm (a) PCL5k (b) PCL10k and c) PCL15k; Figure S3: 1H-NMR spectra of 4-arm (a) PCL5k (b) PCL10k and (c) PCL15k; Figure S4: 1H-NMR spectra of 6-arm (a) PCL5k (b) PCL10k and (c) PCL15k; Figure S5: FTIR spectra of 4-arm (a) PCL5k-PEG, (b) PCL10k-PEG, and (c) PCL15k-PEG; Figure S6: FTIR spectra of 6-arm (a) PCL5k-PEG, (b) PCL10k-PEG, and (c) PCL15k-PEG; Figure S7: 1H-NMR spectra of 4-arm (a) PCL5k-PEG, (b) PCL10k-PEG, and (c) PCL15k-PEG; Figure S8: 1H-NMR spectra of 6-arm (a) PCL5k-PEG, (b) PCL10k-PEG, and (c) PCL15k-PEG; Figure S9: Inhibition zone for *Escherichia coli*; Figure S10: Inhibition zone for Staphylococcus aureus; Table S1: Cumulative release frequency (%) of the polymeric hydrogel formulations; Table S2: Inhibition zone of all the formulations.
