# Supplementary material for: Hydrophobic Drug Carrier from Polycaprolactone-b-Poly(Ethylene Glycol) Star-Shaped Polymers Hydrogel Blend as Potential for Wound Healing Application
Source: Polymers (Basel). 2023 Apr 27;15(9):2072. doi: 10.3390/polym15092072 (PMC10181117; doi:10.3390/polym15092072)
Supplement: Supplementary file 1 [file polymers-15-02072-s001.zip › polymers-2247790-supplementary.pdf]

Title: HYDROPHOBIC DRUG CARRIER FROM POLYCAPROLACTONE-B-POLY(ETHYLENE GLYCOL) STAR-SHAPED POLYMERS HYDROGEL BLEND AS POTENTIAL FOR WOUND HEALING APPLICATION

SUPPLEMENTARY MATERIAL

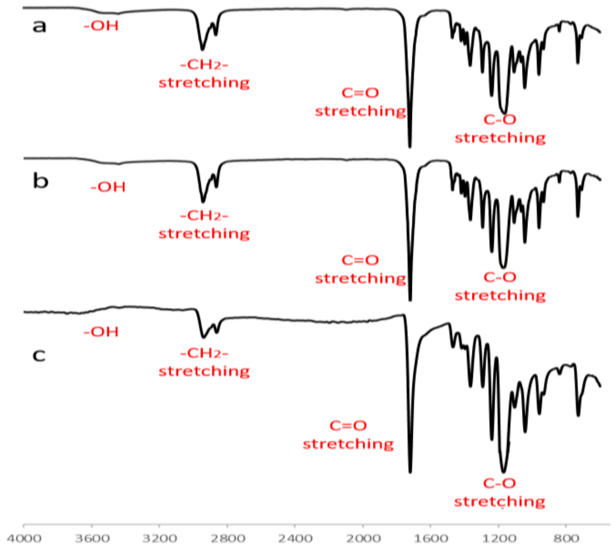

Figure S1. FTIR spectra of 4-arm (**a**) PCL_{5k} (**b**) PCL_{10k} and (**c**) PCL_{15k}.

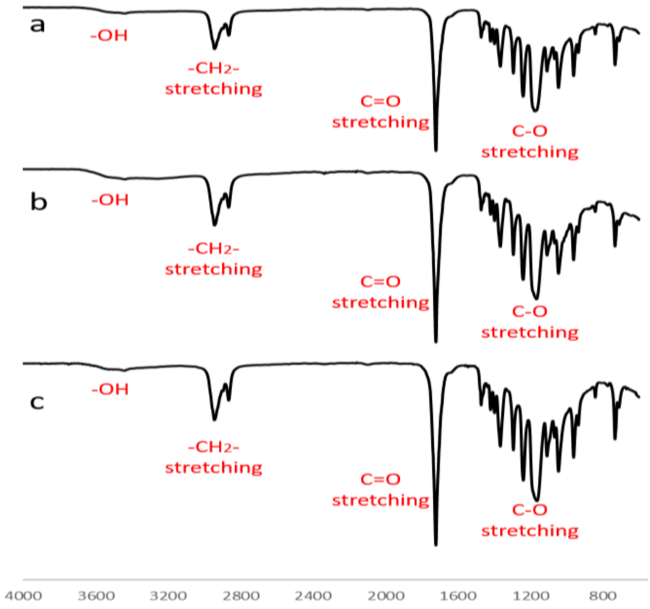

Figure S2. FTIR spectra of 6-arm (**a**) PCL_{5k} (**b**) PCL_{10k} and (**c**) PCL_{15k}.

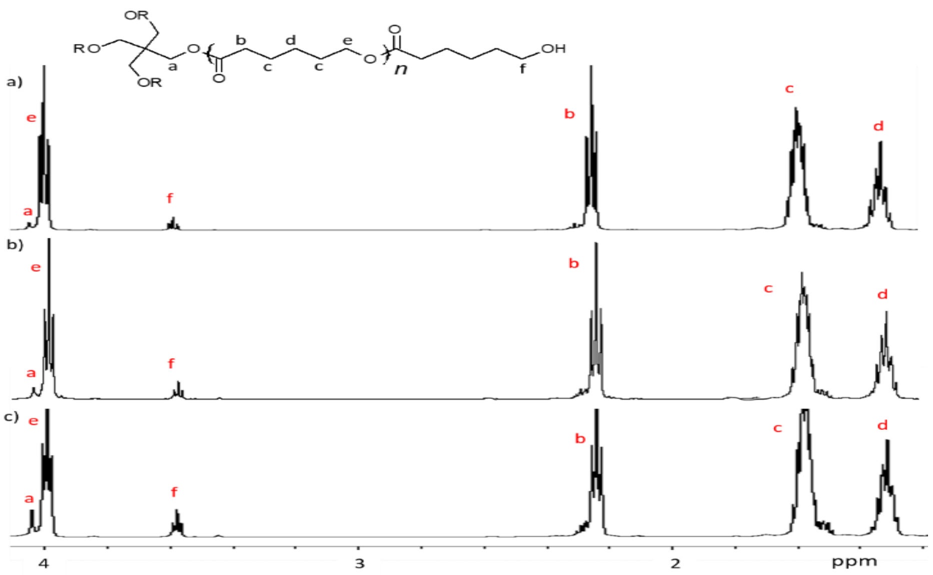

Figure S3. ^{1}H-NMR spectra of 4-arm (**a**) PCL_{5k} (**b**) PCL_{10k} and (**c**) PCL_{15k}.

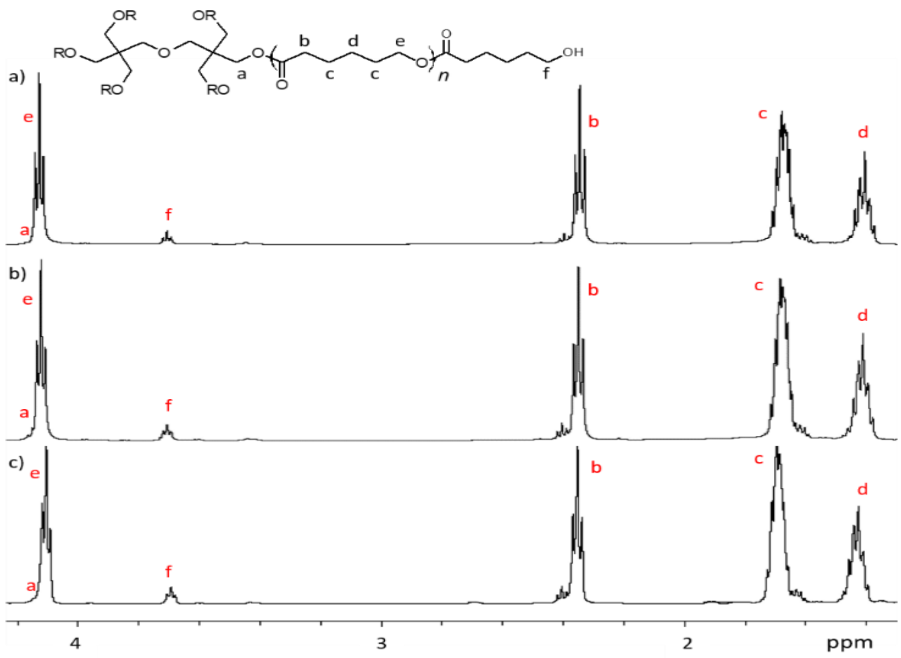

Figure S4. ^{1}H-NMR spectra of 6-arm (**a**) PCL_{5k} (**b**) PCL_{10k} and (**c**) PCL_{15k}.

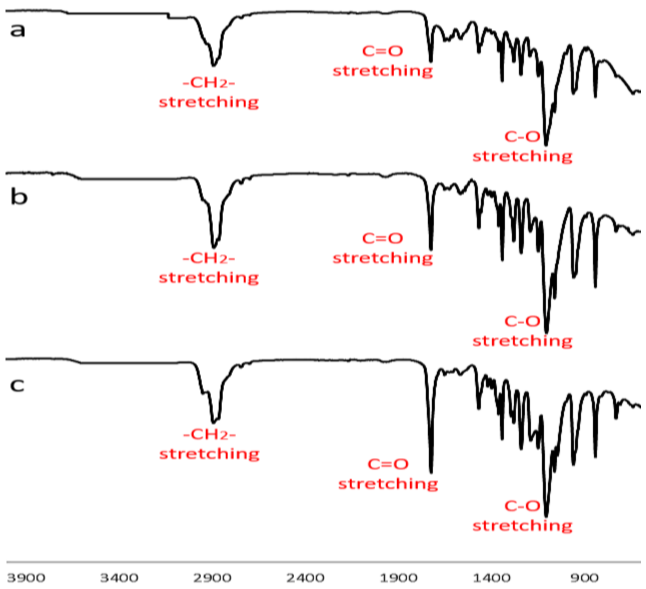

Figure S5. FTIR spectra of 4-arm (**a**) PCL_{5k}-PEG, (**b**) PCL_{10k}-PEG, and (**c**) PCL_{15k}-PEG.

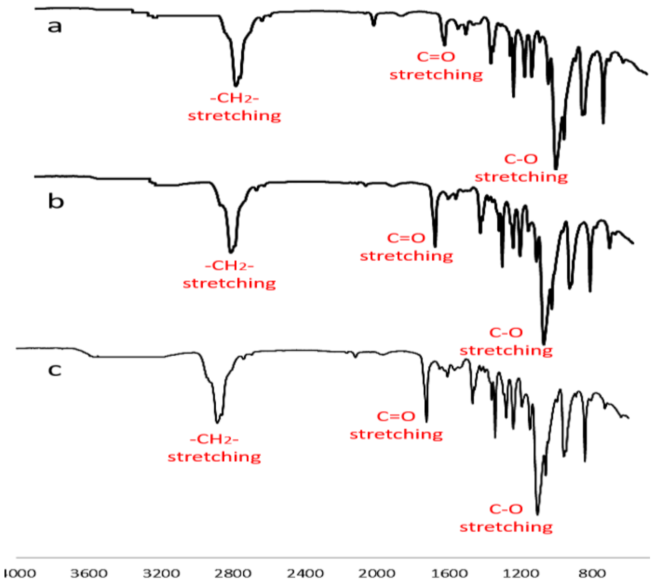

Figure S6. FTIR spectra of 6-arm (**a**) PCL_{5k}-PEG, (**b**) PCL_{10k}-PEG, and (**c**) PCL_{15k}-PEG

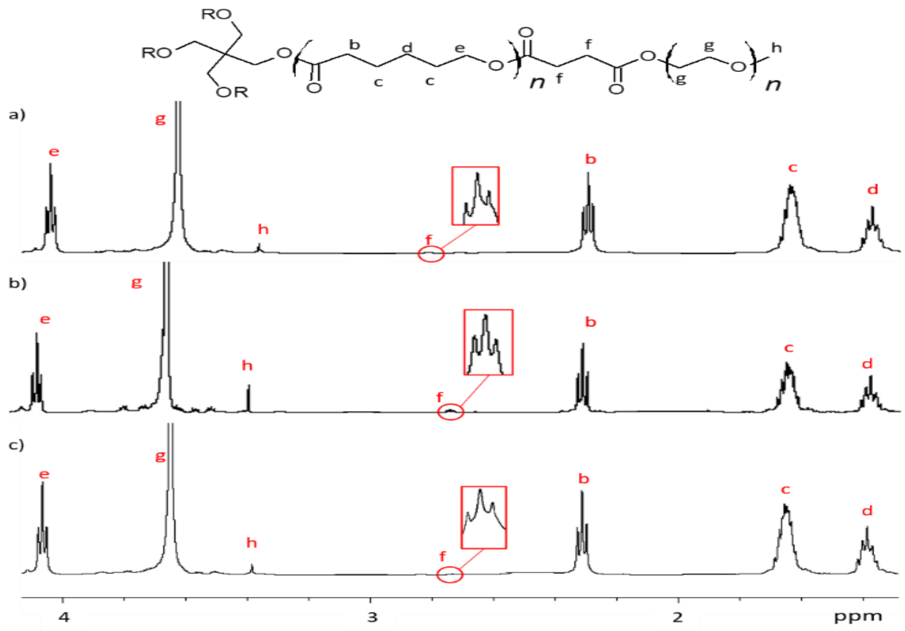

Figure S7. ^{1}H-NMR spectra of 4-arm (**a**) PCL_{5k}-PEG, (**b**) PCL_{10k}-PEG, and (**c**) PCL_{15k}-PEG.

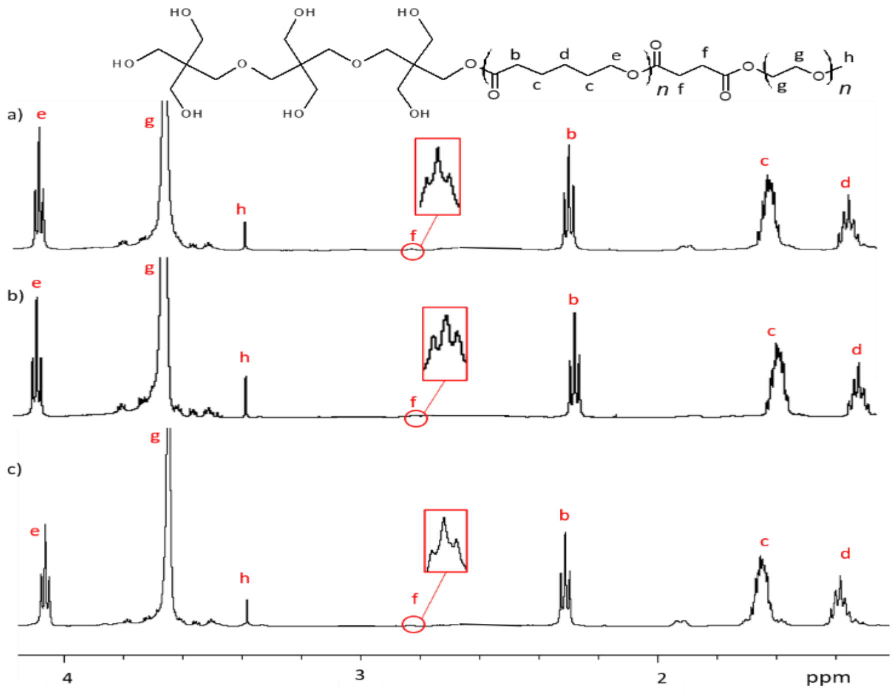

Figure S8. ^{1}H-NMR spectra of 6-arm (**a**) PCL_{5k}-PEG, (**b**) PCL_{10k}-PEG, and (**c**) PCL_{15k}-PEG.

Table S1. Cumulative release frequency (%) of the polymeric hydrogel formulations

Formulation		Cumulative release %						
		1 hr	2 hr	3 hr	4 hr	5hr	6 hr	7 hr
F1	A	0.0110± 0.0044	0.0212± 0.0079	0.0310± 0.0112	0.0406± 0.0144	0.0498± 0.0178	0.0587± 0.0208	0.0674± 0.0238
	B	0.0128± 0.0013	0.0252± 0.0027	0.0374± 0.0039	0.0492± 0.0052	0.0608± 0.0066	0.0722± 0.0080	0.0830±0 .0092
F2	A	0.0149±0 .0013	0.0284±0 .0023	0.0415±0 .0033	0.0539±0 .0042	0.0661±0 .0050	0.0780±0 .0059	0.0896±0 .0066
	B	0.0158±0 .0008	0.0307±0 .0016	0.0452±0 .0023	0.0590±0 .0029	0.0727±0 .0035	0.0861±0 .0043	0.0990±0 .0048
F3	A	0.0162±0 .0017	0.0311±0 .0026	0.0459±0 .0039	0.0602±0 .0055	0.0748±0 .0071	0.0889±0 .0084	0.1019±0 .0100
	B	0.0202±0 .0027	0.0394±0 .0051	0.0578±0 .0077	0.0759±0 .0100	0.0939±0 .0125	0.1116±0 .0147	0.1272±0 .0163
F4	A	0.0127±0 .0007	0.0252±0 .0016	0.0372±0 .0024	0.0489±0 .0033	0.0603±0 .0042	0.0709±0 .0050	0.0811±0 .0057
	B	0.0102±0 .0015	0.0198±0 .0030	0.0290±0 .0044	0.0378±0 .0059	0.0462±0 .0072	0.0542±0 .0085	0.0618±0 .0099
F5	A	0.0143±0 .0034	0.0275±0 .0065	0.0401±0 .0096	0.0527±0 .0138	0.0628±0 .0141	0.0735±0 .0169	0.0837±0 .0199
	B	0.0106±0 .0015	0.0210±0 .0030	0.0311±0 .0043	0.0408±0 .0057	0.0500±0 .0070	0.0590±0 .0083	0.0673±0 .0094
F6	A	0.0150±0 .0009	0.0295±0 .0016	0.0434±0 .0023	0.0568±0 .0030	0.0697±0 .0037	0.0821±0 .0044	0.0938±0 .0051
	B	0.0126±0 .0005	0.0243±0 .0011	0.0353±0 .0016	0.0460±0 .0021	0.0562±0 .0027	0.0657±0 .0031	0.0749±0 .0035

Table S2. Inhibition zone of all the formulations

Formulations		Microorganisms					
		Staphylococcus aureus			*Escherichia coli*		
		Inhibition zone (mm)			Inhibition zone (mm)		
		1 h	12 h	24 h	1 h	12 h	24 h
F3	A	x	9.3	19.0	x	10.0	20.0
	B	x	10.0	20.3	x	10.7	21.0
F6	A	x	9.0	19.0	x	9.3	20.0
	B	x	8.7	18.3	x	7.3	19.0
STD_{F3}	A	x	x	x	x	x	x
	B	x	x	x	x	x	x
STD_{F6}	A	x	x	x	x	x	x
	B	x	x	x	x	x	x

F3 = Formulations with 4-arm PCL_{15k}-PEG and Ciprofloxacin
F6 = Formulations with 6-arm PCL_{15k}-PEG and Ciprofloxacin
STD_{F3} = Formulations with 4-arm PCL_{15k}-PEG
STD_{F6} = Formulations with 6-arm PCL_{15k}-PEG

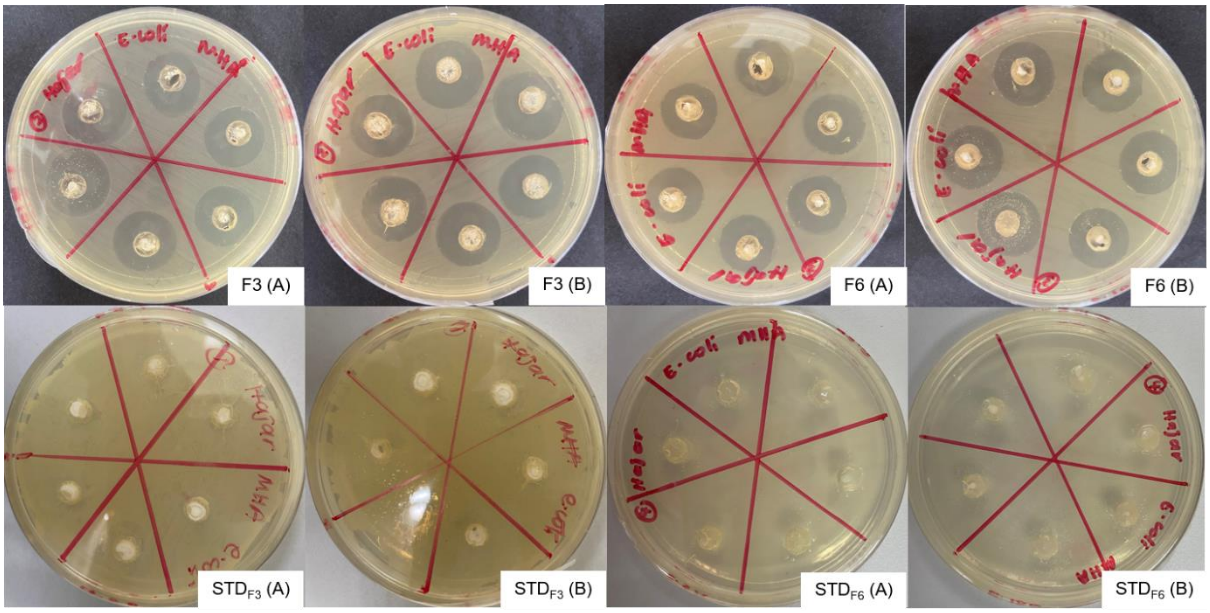

Figure S9. Inhibition zone for *Escherichia coli*

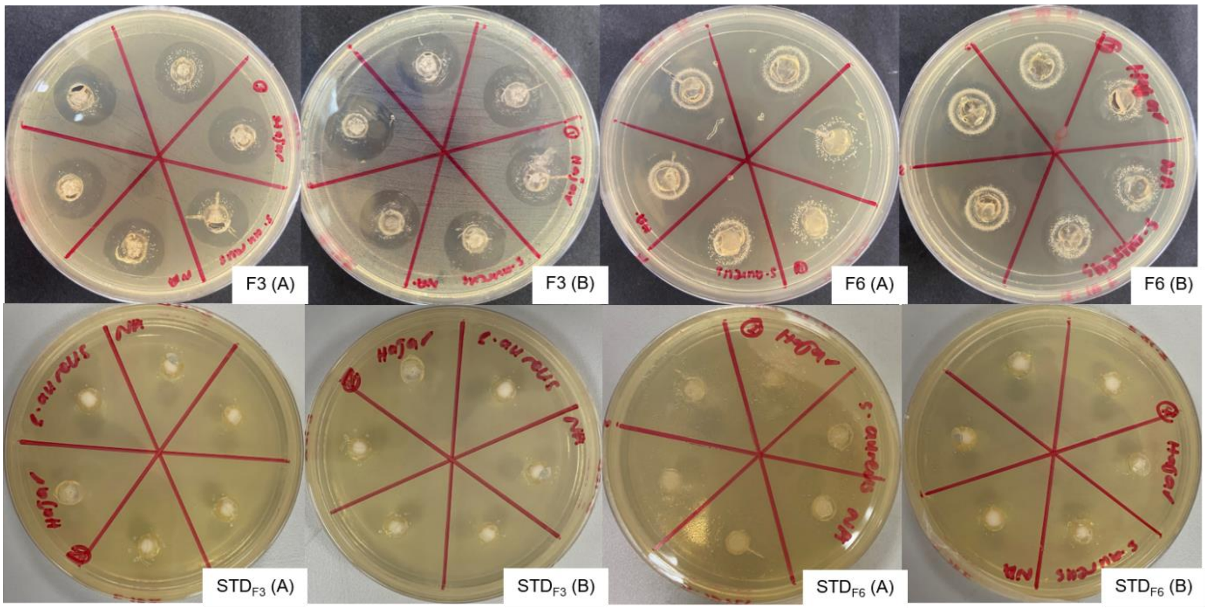

Figure S10. Inhibition zone for *Staphylococcus aureus*
